# Large-scale neuromorphic modeling of cortical networks on FPGA for investigating anesthetic-induced neural dynamics

**DOI:** 10.3389/fnsys.2026.1866947

**Published:** 2026-08-26

**Authors:** Chuanguang Wang, Xiaotian Pan, Si Chen, Hao Cheng, YuJie Yao, Guodao Zhang, Fang Zheng

**Affiliations:** 1 Department of Anesthesia, Lishui Municipal Central Hospital, Lishui, Zhejiang, China; 2 Department of Anesthesia, The Fifth Affiliated Hospital of Wenzhou Medical University, Lishui, China; 3 Institute of Intelligent Media Computing, Hangzhou Dianzi University, Hangzhou, Zhejiang, China

**Keywords:** field-programmable gate array (FPGA), general anesthesia, Izhikevich model, neuromorphic engineering, real-time simulation, spiking neural networks (SNN)

## Abstract

**Introduction:**

Understanding the neural mechanisms underlying general anesthesia remains a significant challenge in neuroscience and clinical practice. Traditional software-based simulations of large-scale brain networks are often constrained by high computational costs and fail to achieve real-time performance.

**Methods:**

In this paper, we propose a high-performance hardware implementation of large-scale neuromorphic system to investigate anesthetic-induced neural dynamics. The system successfully models a cortical network comprising 10,000 spiking neurons (8,000 excitatory and 2,000 inhibitory) utilizing the biologically plausible Izhikevich neuron model. Deployed on a field-programmable gate array (FPGA), the proposed architecture exploits high parallelism to achieve real-time simulation speeds. By adjusting synaptic weights and network parameters to mimic the pharmacological eects of anesthetic agents, our system can continuously monitor and evaluate state transitions in neural synchronization and firing patterns.

**Results:**

The results demonstrate that the hardware-accelerated neuromorphic approach provides an efficient, scalable, and real-time platform for investigating large-scale neural dynamics.

**Discussion:**

Pending future validation against empirical clinical EEG data, this foundational framework paves the way for advanced brain–machine interfaces and closed-loop anesthetic delivery systems.

## 1 Introduction

General anesthesia is a complex pharmacological intervention characterized by the reversible loss of consciousness, amnesia, and immobility. Despite its widespread clinical use, the precise neurobiological mechanisms underlying the transition from the awake state to deep anesthesia remain an active area of research (Brown et al., 2010). At the microscopic level, anesthetic agents typically modulate synaptic transmission, often by enhancing GABAergic inhibition or suppressing glutamatergic excitation (Campagna et al., 2003). These cellular-level alterations profoundly impact macroscopic brain dynamics, leading to characteristic changes in population firing rates, neural synchronization, and macroscopic rhythms traditionally observed in electroencephalography (EEG) (Ching et al., 2010, 2012; Purdon et al., 2013). Consequently, investigating how local synaptic disruptions propagate to cause global brain state transitions requires a robust computational framework.

To bridge the gap between microscopic cellular mechanisms and macroscopic behavioral states, computational modeling of neural networks is essential. Spiking neural networks (SNNs) provide a biologically plausible environment to study the spatiotemporal dynamics of the brain (Einevoll et al., 2019; Destexhe et al., 2003). By defining specific neuron models (such as integrate-and-fire or Izhikevich models) and incorporating realistic synaptic kinetics, researchers can simulate the effects of anesthetic agents on network behavior. Large-scale SNN modeling is particularly crucial, as the emergent phenomena associated with anesthesia—such as the collapse of neural complexity and the shift in dominant frequency bands—cannot be accurately captured by isolated single-neuron simulations (Mashour and Hudetz, 2019). A network approach allows for the observation of collective dynamics, population firing rates, and the continuous state transitions corresponding to different depths of anesthesia (e.g., light and deep anesthesia).

Traditionally, large-scale SNN models for anesthesia are simulated using software environments executed on general-purpose CPUs or GPUs (Majidifar et al., 2025; Moriya et al., 2025; Heidarpur et al., 2019). While software simulations offer high precision and flexibility, they suffer from significant drawbacks, including high power consumption, inherent sequential processing bottlenecks, and the inability to achieve true real-time performance for massive networks (Majidifar et al., 2022). To overcome these limitations, neuromorphic hardware implementations have emerged as a powerful alternative (Majidifar et al., 2023; Nazari and Jamshidi, 2024; Sun et al., 2025). Field-programmable gate arrays (FPGAs), in particular, offer highly parallelized architectures, deterministic timing, low latency, and exceptional energy efficiency (Leigh et al., 2023; Chen et al., 2024; Shama et al., 2020; Zhang et al., 2023; Ghanbarpour et al., 2023). By mapping the highly interconnected structure of biological neural networks directly onto digital hardware, FPGAs enable the real-time emulation of complex brain states, making them highly suitable for advanced brain-machine interfaces and closed-loop anesthetic delivery systems (Heidarpur et al., 2020; Jokar et al., 2019).

While neuromorphic models of brain states have been explored, the real-time emulation of large-scale networks under distinct pharmacological conditions like anesthesia presents unique challenges in balancing biological realism, computational scale, and hardware efficiency. This paper addresses these challenges by introducing an FPGA-based architecture for modeling anesthetic-induced brain dynamics. Our work is distinguished by its scale and rigorous validation, offering a significant advancement in hardware-based computational neuroscience.

The main contributions of this work are summarized as follows:

1. A scalable and real-time neuromorphic architecture: We present a resource-optimized digital architecture capable of the real-time emulation of a 10,000-neuron spiking network. The design is specifically tailored to capture the complex, emergent dynamics that are only observable at such a significant scale, providing a powerful platform for large-scale brain simulations.
2. High-fidelity hardware model of anesthesia: We demonstrate a hardware-level implementation of a pharmacodynamic model, successfully replicating the hallmark neurophysiological transitions from wakefulness to deep anesthesia. By dynamically modulating synaptic and neuronal parameters, our model accurately reproduces key biomarkers of anesthesia, such as suppressed population firing rates and altered neural synchrony.
3. Bridging the gap between precision and efficiency: We establish a rigorous validation framework demonstrating that our optimized fixed-point hardware implementation achieves near-perfect correlation (*r*>0.98) with its floating-point software counterpart. This confirms the architecture's computational fidelity at both macroscopic (population dynamics) and microscopic (single-neuron voltage) levels, proving that hardware efficiency can be achieved without sacrificing scientific accuracy.

The remainder of this paper is organized as follows: Section 2 details the mathematical modeling of the large-scale neuromorphic network. Section 3 presents the network-level mechanisms and dynamic phase transitions of anesthesia. Section 4 elaborates on the digital hardware implementation, resource utilization, and digital validation on the FPGA. Finally, Section 5 concludes the paper.

## 2 Modeling the large-scale neuromorphic network for anesthesiology

To investigate the macroscopic phenomena of general anesthesia (such as burst suppression and loss of consciousness) via an *in-silico* hardware framework, a highly scalable, biologically plausible neural network is essential. In this section, we formulate the mathematical foundation of a large-scale spiking neural network (SNN) based on the Izhikevich neuron model (Izhikevich, 2003). The network is explicitly designed to mimic the structural connectivity of the mammalian cortex, providing a rigorous substrate for linking cellular-level synaptic modulations to network-wide clinical anesthesiology markers.

### 2.1 Anesthesiology mechanisms and the neuromorphic paradigm

General anesthesia is a pharmacological intervention that induces a reversible loss of consciousness, amnesia, and analgesia (Hudetz, 2012). From a neurobiological perspective, anesthetic agents, such as propofol or isoflurane, do not uniformly disable neurons; rather, they disrupt the balance of excitation and inhibition (*E*/*I* balance) within the brain (Campagna et al., 2003; Mashour and Hudetz, 2019). Most widely used intravenous anesthetics act primarily as positive allosteric modulators of *GABA*_*A*_ receptors (Brown et al., 2010; Campagna et al., 2003), effectively prolonging the duration of inhibitory postsynaptic currents (IPSCs) and hyperpolarizing target neurons. Conversely, some agents act by blocking excitatory NMDA receptors.

Studying these complex transitions requires simulating thousands to millions of interconnected neurons. Traditional software-based simulations face severe bottlenecks in executing large-scale, highly connected differential equations in real-time. Neuromorphic engineering, particularly FPGA-based implementations, offers a paradigm shift. Using massive parallelism and dedicated digital architectures, FPGAs can simulate large-scale cortical networks with biological time-scales, enabling the direct observation of anesthetic-induced rhythmic alterations—such as the transition from active awake states to slow-wave activity and profound burst suppression—in real-time hardware.

### 2.2 Mathematical formulation of the 10,000-neuron synaptic network

To balance biological realism with hardware efficiency, the network is constructed using *N* = 10,000 spiking neurons. Following the biological composition of the neocortex (Markram et al., 2004), the population is divided into a ratio of 4:1, comprising *N*_*E*_ = 8,000 excitatory neurons and *N*_*I*_ = 2,000 inhibitory neurons.

#### 2.2.1 Neuronal dynamics

The intrinsic dynamics of each neuron *i* (where *i* ∈ {1, 2, …, *N*}) are governed by the Izhikevich model, which uses a two-dimensional system of ordinary differential equations. The state of neuron *i* is defined by its membrane potential, *v*_*i*_(*t*), and a slow recovery variable, *u*_*i*_(*t*):


$$\begin{array}{l} \frac{dv_{i}}{dt}=0.04v_{i}^{2}+5v_{i}+140-u_{i}+I_{i}^{syn}\left(t\right)+I_{i}^{bg}\left(t\right) \end{array}$$ (1)

$$\begin{array}{l} \frac{du_{i}}{dt}=a_{i}\left(b_{i}v_{i}-u_{i}\right) \end{array}$$ (2)

with the discrete spike-reset condition:


$$\begin{array}{l} \text{if }v_{i}\geq 30\text{ mV, then}\{\begin{array}{l} v_{i}\leftarrow c_{i}\\ u_{i}\leftarrow u_{i}+d_{i} \end{array} \end{array}$$ (3)

Here, $I_{i}^{syn}\left(t\right)$ represents the total synaptic current received from the network, and $I_{i}^{bg}\left(t\right)$ is an external background noise current simulating subcortical (e.g., thalamic) inputs. The parameters *a*_*i*_, *b*_*i*_, *c*_*i*_, and *d*_*i*_ determine the specific firing patterns. For the excitatory population, parameters are tuned to exhibit Regular Spiking (RS) behavior, while the inhibitory population is configured for fast spiking (FS) dynamics, accurately reflecting cortical cell types.

#### 2.2.2 Realistic cortical topology and connectivity matrix

A purely random or all-to-all connectivity does not reflect the structural reality of the brain. Instead, the mammalian cortex features a spatially embedded, distance-dependent topology that minimizes wiring length while maintaining integration.

We map the *N* neurons onto a 2D spatial grid. The physical distance between neuron *i* and neuron *j* is denoted as *D*(*i, j*). The structural connectivity is defined by an asymmetric adjacency matrix **A** ∈ {0, 1}^*N*×*N*^, where *A*_*ij*_ = 1 indicates a directed synaptic connection from presynaptic neuron *j* to postsynaptic neuron *i*. The probability of a connection is modeled as an exponentially decaying function of distance:


$$\begin{array}{l} P\left(A_{ij}=1\right)=C_{prob}\cdot \exp\left(-\frac{D\left(i,j\right)}{\lambda }\right) \end{array}$$ (4)

where λ is the characteristic spatial length of the axonal arborization, and *C*_*prob*_ is the base connection probability. To strictly accommodate the entire connectivity matrix within the FPGA's on-chip Block RAMs and bypass the severe latency bottlenecks of external DRAM, the spatial decay constraints were aggressively bounded. This offline generation yielded a highly localized, sparse topology comprising exactly 68,608 physical synapses across the 10,000 neurons, equating to an average hardware fan-out of ≈6.86 synapses per neuron.

The corresponding weight matrix **W** ∈ ℝ^*N*×*N*^ is formulated as Equation 5:


$$\begin{array}{l} W_{ij}=A_{ij}\times w_{j} \end{array}$$ (5)

where *w*_*j*_ > 0 if *j* ∈ *N*_*E*_ (glutamatergic synapse), and *w*_*j*_ < 0 if *j* ∈ *N*_*I*_ (GABAergic synapse).

To establish the distance-dependent synaptic connectivity and maintain the network's spontaneous activity, two fundamental formulations are integrated into the model. First, the one-dimensional neuron indices *i, j* ∈ {0, 1, …, *N*−1} are mapped onto a two-dimensional logical grid of size *L*×*L* (where $L=\sqrt{N}$). The coordinates are defined as *x*_*i*_ = *i* (mod *L*) and *y*_*i*_ = ⌊*i*/*L*⌋, yielding the Euclidean distance $D\left(i,j\right)=\sqrt{{\left(x_{i}-x_{j}\right)}^{2}+{\left(y_{i}-y_{j}\right)}^{2}}$. This logical distance is evaluated entirely offline to generate the sparse adjacency matrix, allowing the connections to be stored efficiently in FPGA Block RAMs (BRAMs) to prevent physical routing congestion. Second, to sustain the spontaneous firing state essential for observing macroscopic patterns, a background current $I_{i}^{bg}\left(t\right)=I_{bias}+\sigma \xi_{i}\left(t\right)$ is injected into each neuron. Here, *I*_*bias*_ is a constant driving baseline and $\xi_{i}\left(t\right)\sim N\left(0,1\right)$ represents standard Gaussian noise. To preserve a multiplier-less architecture and minimize Digital Signal Processing (DSP) slice utilization, the stochastic term is efficiently approximated on the FPGA using linear feedback shift registers (LFSRs) combined with logic shift operations, bypassing the need for resource-intensive random number generators.

It is crucial to acknowledge that general anesthesia is fundamentally a whole-brain phenomenon involving profound thalamocortical loop interactions (Brown et al., 2010; Ching et al., 2010). However, strictly constrained by the on-chip memory limits of the FPGA, explicitly instantiating separate thalamic populations was unfeasible. Therefore, our architecture represents an isolated intracortical network. The external background current (*I*_*bg*_) utilized in this model does not represent structured thalamocortical input, but rather acts merely as a non-specific excitatory mathematical drive required to sustain spontaneous activity within the isolated patch. Consequently, as simulated anesthesia deepens, the systematic reduction of *I*_*bias*_ strictly abstracts a generalized loss of overall excitatory tone within the isolated cortex, limiting our observations to intracortical dynamics rather than whole-brain phenomena.

#### 2.2.3 Synaptic current and first-order kinetics

To capture the temporal dynamics of synaptic transmission—which is the primary target of anesthetic drugs—we utilize a first-order kinetic model for the synaptic conductance. The total synaptic current arriving at neuron *i* is the linear sum of all active incoming presynaptic connections:


$$\begin{array}{l} I_{i}^{syn}\left(t\right)=\overset{N}{\underset{j=1}{\sum }}W_{ij}g_{j}\left(t\right) \end{array}$$ (6)

The synaptic gating variable *g*_*j*_(*t*) for presynaptic neuron *j* increases instantaneously upon a spike and decays exponentially over time. It is governed by the differential equation:


$$\begin{array}{l} \frac{dg_{j}}{dt}=-\frac{g_{j}\left(t\right)}{\tau_{j}}+\underset{k}{\sum }\delta \left(t-t_{j}^{\left(k\right)}-\Delta t_{delay}\right) \end{array}$$ (7)

where $t_{j}^{\left(k\right)}$ represents the time of the *k*-th spike of neuron *j*, δ(·) is the Dirac delta function, Δ*t*_*delay*_ is the axonal conduction delay, and τ_*j*_ is the synaptic decay time constant.

Crucially, in our proposed framework, the variables defining the inhibitory synapses (τ_*I*_ and the absolute magnitude of *W*_*I*_) will serve as the mathematical interface for modeling anesthesiology. Modulating these parameters allows the hardware network to transition seamlessly into anesthetic-induced states, which will be discussed in the subsequent sections concerning network dynamics.

## 3 Network-level mechanisms of anesthesia: a neuromorphic perspective

General anesthesia represents a pharmacologically induced, reversible loss of consciousness. Rather than uniformly silencing neuronal activity, anesthetic agents such as propofol generally operate by modulating the excitation-inhibition (*E*/*I*) balance within the cerebral cortex (Brown et al., 2010; Campagna et al., 2003). At the synaptic level, this is predominantly achieved via the positive allosteric modulation of *GABA*_*A*_ receptors, which prolongs the decay time constant of inhibitory postsynaptic currents (IPSCs) and increases inhibitory synaptic efficacy. To investigate these transitions within our neuromorphic framework, we modulated the inhibitory time constant (τ_*I*_) and the absolute magnitude of inhibitory synaptic weights (|*W*_*I*_|) to emulate the macroscopic effects of varying anesthetic concentrations.

### 3.1 Simulated anesthetic phases and network dynamics

**Figure 1:**
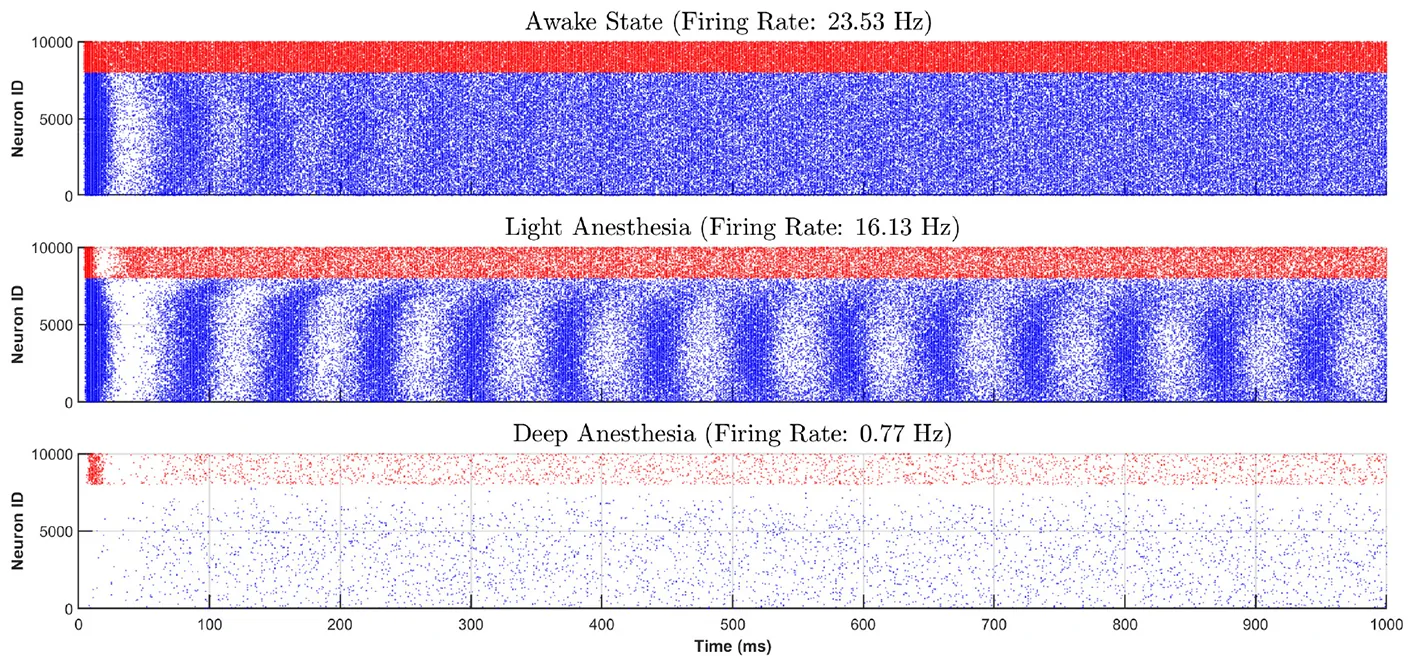
Spatiotemporal raster plots demonstrating the network's transition through simulated anesthetic phases. Blue dots represent excitatory neurons (*N*_*E*_ = 8,000) and red dots denote inhibitory neurons (*N*_*I*_ = 2,000). **(Top)** The awake state exhibits high-frequency, asynchronous firing characteristics of an active cortex. **(Middle)** Light anesthesia reveals synchronous oscillatory dynamics (slow-wave activity) driven by enhanced inhibitory decay time (τ_*I*_). **(Bottom)** Deep anesthesia results in a profoundly suppressed network state due to strong synthetic GABAergic modulation, drastically reducing the overall firing rate.

To validate the model's capability of reproducing biologically plausible macro-states, the simulated network was subjected to three distinct parameterized phases corresponding to varying depths of anesthesia: the awake state, light anesthesia, and deep anesthesia. The network dynamics for these phases are visualized using raster plots of spiking activity across the 10,000-neuron population (Figure 1).

**Figure 2:**
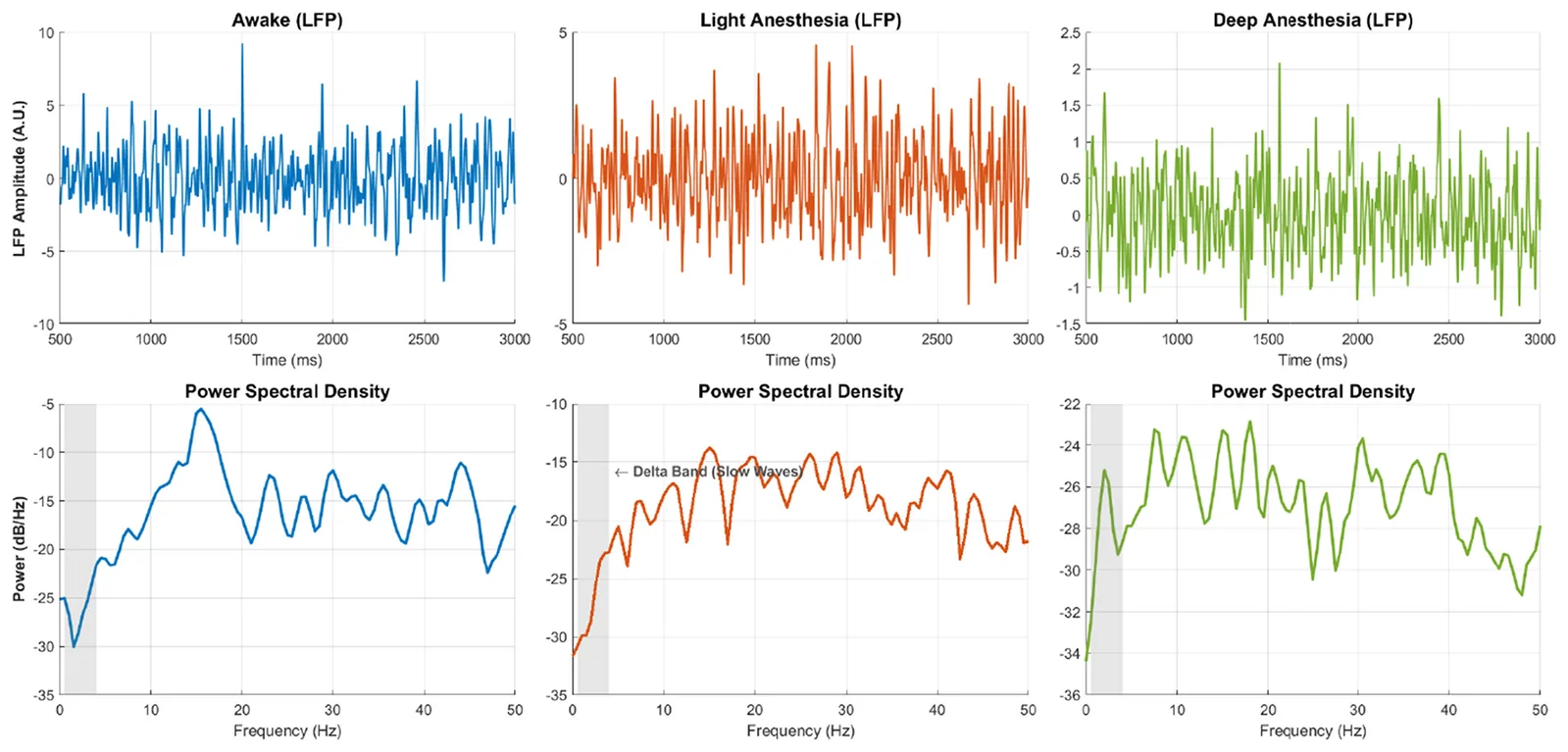
LFP dynamics and power spectral characteristics across anesthesia phases. **(a–c)** Time-domain surrogate LFP signals for awake, light anesthesia, and deep anesthesia states, respectively. **(d–f)** Corresponding power spectral density (PSD) estimates obtained using Welch's method. The shaded region indicates the delta frequency band (0.5–4 Hz), associated with slow-wave activity.

1. Awake state: In the baseline condition, the network operates with a natural E/I balance (τ_*I*_ = 1 ms, nominal |*W*_*I*_|). This phase is characterized by a high firing rate and asynchronous, dense spiking activity (Destexhe et al., 2003), reflecting the active cortical processing and complex integration typically observed in a conscious brain.

2. Light anesthesia (slow-wave synchronization): To mimic the known pharmacological mechanisms of anesthetics on GABAergic synapses (Ching et al., 2010; Hindriks and van Putten, 2012), as the simulated dosage increases, our empirically tuned inhibitory time constant is prolonged (τ_*I*_ = 35 ms) and the inhibitory synaptic factor is amplified by 1.8 × . This profound enhancement of GABAergic influence forces the network out of its asynchronous state and into a highly synchronized, oscillatory regime. The raster plot reveals distinct vertical bands of collective firing, effectively mimicking the macroscopic slow-wave dynamics that are a hallmark of light general anesthesia as observed in intracranial LFP recordings (Sellers et al., 2013; Lewis et al., 2012). To appropriately limit our claims regarding clinical neurophysiology, we explicitly note that our point-neuron network generates a surrogate Local Field Potential (LFP) proxy to track these states, rather than true macroscopic clinical EEG. Furthermore, the biological hallmark of this state transition—the shift of frequency power-bands from high-frequency activity to a low-frequency dominant spectrum—is explicitly quantified via spectral data in Figure 2.

3. Deep anesthesia (profound inactivity): In the deepest simulated state, extreme inhibitory modulation is applied (τ_*I*_ = 100 ms, 3.5 × inhibitory factor) alongside a reduction in background sensory drive. This leads to profound network suppression, where the intense synaptic inhibition overcomes nearly all spontaneous excitatory activity. The network exhibits an extremely sparse firing pattern (rate < 1 Hz), representing the severe attenuation of cortical dynamics necessary for major surgical interventions (Brown et al., 2010; Ching et al., 2012).

As presented in Table 1, stepping through these parameters reveals a continuous macroscopic state transition within the neuromorphic population, characterized by a monotonic decrease in the global firing rate (Mashour and Hudetz, 2019). It is important to note a methodological distinction here: while Table 1 maps a continuous dose-dependent trajectory, the corresponding frequency-domain shifts are evaluated as discrete steady-state snapshots (as subsequently shown in Figure 2). Because our surrogate LFP extraction requires discarding transient initialization artifacts, demonstrating a strictly continuous dynamic phase transition (e.g., via a continuous spectrogram) remains beyond the current evaluation protocol. Thus, to appropriately limit our conclusions, we define this progression as a continuous state transition rather than an abrupt phase shift. In the initial steps (Steps 1–4), which correspond to a baseline or low synthetic anesthetic concentration, τ_*I*_ remains near its physiological baseline (1 ≤ τ_*I*_ < 20 ms). In this regime, the network maintains a robust, asynchronous firing rate (ranging from 18.38 to 23.53 Hz). We explicitly note that this elevated firing rate does not represent a biophysically realistic awake state; empirical *in vivo* data from mammalian cortices demonstrate highly sparse firing in the range of 2–5 Hz (Wei et al., 2023). Rather, our 20 Hz baseline is a mathematically necessitated “synthetic asynchronous state.” Due to the extreme synaptic sparsity (≈6.86 synapses per neuron) required to strictly fit the model within FPGA memory limits, this artificially elevated excitability is mandatory to sustain network criticality and prevent the complete collapse of spontaneous activity.

**Table 1:** Continuous parametric sweep simulating the progression of anesthesia.

Step	τ_*I*_ (ms)	*W*_*factor*_	*I*_*bias*_	σ	Firing rate (Hz)	Estimated phase
1	1.00	1.00	5.00	5.00	23.53	Awake
2	6.21	1.13	4.79	4.87	21.73	Awake
3	11.42	1.26	4.58	4.74	19.99	Awake
4	16.63	1.39	4.37	4.61	18.38	Awake
5	21.84	1.53	4.16	4.47	16.82	Light anesthesia (slow-wave)
6	27.05	1.66	3.95	4.34	15.42	Light anesthesia (slow-wave)
7	32.26	1.79	3.74	4.21	14.16	Light anesthesia (slow-wave)
8	37.47	1.92	3.53	4.08	12.97	Light anesthesia (slow-wave)
9	42.68	2.05	3.32	3.95	11.92	Light anesthesia (slow-wave)
10	47.89	2.18	3.11	3.82	10.86	Light anesthesia (slow-wave)
11	53.11	2.32	2.89	3.68	9.89	Light anesthesia (slow-wave)
12	58.32	2.45	2.68	3.55	8.91	Light anesthesia (slow-wave)
13	63.53	2.58	2.47	3.42	7.93	Light anesthesia (slow-wave)
14	68.74	2.71	2.26	3.29	6.93	Light anesthesia (slow-wave)
15	73.95	2.84	2.05	3.16	5.92	Deep anesthesia
16	79.16	2.97	1.84	3.03	4.82	Deep anesthesia
17	84.37	3.11	1.63	2.89	3.63	Deep anesthesia
18	89.58	3.24	1.42	2.76	2.46	Deep anesthesia
19	94.79	3.37	1.21	2.63	1.46	Deep anesthesia
20	100.00	3.50	1.00	2.50	0.79	Deep anesthesia

As the simulated anesthetic dosage increases (Steps 5–14), the prolonged decay of inhibitory currents (21.84 ≤ τ_*I*_ ≤ 68.74 ms) forces the network into an oscillatory regime. This intermediate phase, labeled as Light Anesthesia, represents a bifurcation in the network dynamics where recurrent inhibition periodically synchronizes and silences the neuronal population, manifesting as slow-wave activity. Consequently, the mean firing rate gradually decays from 16.82 Hz down to 6.93 Hz.

Ultimately, at high simulated concentrations (steps 15–20), the overwhelming inhibitory efficacy (τ_*I*_≥73.95 ms, *W*_*factor*_≥2.84) combined with a significantly reduced excitatory background drive drastically attenuates spontaneous spiking. The network enters the Deep Anesthesia phase, where the firing rate continues its steady decline from approximately 5.92 Hz down to a state of profound suppression, eventually falling below 1 Hz (0.79 Hz at step 20). This parametric progression effectively validates the model's ability to replicate the graded pharmacological effects of anesthetics, demonstrating a biologically plausible dose-response relationship in hardware-oriented spiking neural networks.

To rigorously validate these macroscopic state transitions, the neurophysiological frequency shifts explicitly requested by biological standards—specifically the attenuation of high-frequency power and the emergence of a dominant low-frequency delta band (0.5–4 Hz) during simulated anesthesia—are quantitatively evaluated using surrogate LFP power spectral density (PSD) data in Section 3.2 and Figure 2. This methodological separation ensures that while Figure 1 visualizes the spatiotemporal spike timings, the macroscopic spectral hallmarks are robustly validated with frequency-domain data.

### 3.2 LFP and power spectral analysis across anesthesia phases

To further investigate the macroscopic network dynamics under different anesthetic conditions, we analyzed the simulated neural activity in terms of surrogate local field potentials (LFP) and their corresponding power spectral density (PSD). LFP signals provide a coarse-grained representation of collective neuronal activity and are commonly used in both experimental and computational neuroscience to characterize large-scale cortical dynamics.

In the present model, the surrogate LFP was computed as the temporally smoothed population spike count across the network. It is important to acknowledge that this approach serves as a coarse-grained proxy for macroscopic network activity. While it effectively captures population firing rhythms and synchronization in point-neuron networks, it is not equivalent to a true biophysical LFP, which is fundamentally driven by spatially integrated transmembrane and synaptic currents in compartmental morphologies. Despite this limitation, computing surrogate LFP from integrate-and-fire network models is a validated approach (Mazzoni et al., 2015), and we utilize this mesoscopic metric to track the large-scale state transitions induced by our synthetic anesthetic modulation. To remove initialization artifacts, the first 500 ms of simulation were discarded and the remaining signals were used for spectral analysis.

Figure 2 illustrates the LFP time series and corresponding PSD estimates for the three simulated brain states: awake, light anesthesia, and deep anesthesia. The parameters used in the simulations are summarized in Table 2.

**Table 2:** Simulation parameters used for modeling anesthesia-induced network dynamics.

Parameter	Value
Number of neurons (*N*)	10,000
Excitatory neurons (*N*_*E*_)	8,000 (80%)
Inhibitory neurons (*N*_*I*_)	2,000 (20%)
Simulation time	3,000 ms
Time step (*dt*)	1 ms
Base connection probability (*C*_*prob*_)	0.1
Spatial decay constant (λ)	3.4904
Excitatory weight (*w*_*ex*_)	0.5
Inhibitory weight (*w*_*in*_)	−1.0
Excitatory synaptic time constant (τ_*E*_)	5 ms

The first row of Figure 2 shows the time-domain surrogate LFP signals. In the awake state (Figure 2a), the surrogate LFP exhibits irregular, high-variability fluctuations reflecting asynchronous neuronal activity. Such desynchronized dynamics are consistent with experimentally observed local field potentials during active wakefulness.

During light anesthesia (Figure 2b), the network begins to exhibit more structured fluctuations with increased low-frequency components. This transition reflects the emergence of partially synchronized neuronal populations caused by increased inhibitory influence and reduced background excitation.

In the deep anesthesia state (Figure 2c), the LFP amplitude becomes more regular and slower fluctuations dominate the signal. These dynamics correspond to strongly synchronized neuronal firing patterns typically associated with anesthetic-induced slow oscillations at the mesoscopic LFP level (Sellers et al., 2013; Lewis et al., 2012).

The second row of Figure 2 presents the power spectral density of the corresponding LFP signals. In the Awake state (Figure 2d), the power spectrum is relatively broad without a pronounced low-frequency peak, indicating desynchronized cortical activity. Under Light Anesthesia (Figure 2e), power in the delta band (0.5–4 Hz) begins to increase, suggesting the onset of slow-wave activity. This effect becomes more prominent in the deep anesthesia state (Figure 2f), where the spectral power is further concentrated in the low-frequency range, reflecting the dominance of slow oscillations.

The shaded region in the PSD plots highlights the delta frequency band (0.5–4 Hz), which is widely recognized as a key spectral marker of anesthetic depth representing synchronized local cortical down-states (Sellers et al., 2013; Lewis et al., 2012).

Overall, these results demonstrate that the proposed spiking neural network model reproduces key qualitative features of cortical activity observed across different anesthesia levels, including the transition from asynchronous high-frequency activity to synchronized slow-wave dynamics.

To explicitly quantify these anesthetic-induced spectral shifts, we calculated the relative signal power within the delta frequency band (0.5–4Hz) against the total spectrum (0.5–50Hz). In the highly active Awake state, the relative delta-band power accounted for merely 0.01% of the total spectral power. As the network transitioned into light anesthesia, the profound enhancement of GABAergic influence synchronized the network, causing the relative delta power to significantly surge to 2.86%. Finally, in the Deep Anesthesia phase, the low-frequency slow waves became highly prominent within the heavily suppressed network, with the delta band's relative contribution increasing to 8.28% of the spectral power. These quantitative metrics robustly corroborate the visual PSD estimates and closely mirror the spectral trends clinically observed during general anesthesia.

It is important to explicitly acknowledge a structural limitation regarding the spectral signatures of the light anesthesia state. While our model successfully captures the progression of slow-wave dynamics, the surrogate LFP in Figure 2 does not reproduce the prominent alpha-band (8–12 Hz) peak typically observed in clinical EEG during light anesthesia. Biologically, anesthesia-induced alpha rhythms are predominantly generated by thalamocortical loop interactions. Because our current neuromorphic architecture implements an isolated intracortical network without subcortical thalamic inputs, the generation of these specific alpha oscillations is inherently precluded. Consequently, the biophysical conclusions drawn from this model must be appropriately limited to intracortical dynamic shifts, rather than whole-brain thalamocortical phenomena.

### 3.3 Breakdown of critical dynamics and neuronal avalanches

To investigate the macroscopic transitions of the brain state under anesthesia from a statistical physics perspective, we analyzed the network's behavior within the framework of criticality. Accumulating evidence suggests that the awake mammalian cortex operates near a critical phase transition, an optimal state that maximizes information processing, dynamic range, and memory storage. A hallmark of this critical state is the emergence of “neuronal avalanches”—spatiotemporal cascades of spontaneous spiking activity (Beggs and Plenz, 2003). In a critical regime, the probability distribution of avalanche sizes (*S*) lacks a characteristic scale and robustly follows a power-law distribution:


$$P\left(S\right)\propto S^{-\alpha }$$

where empirical and theoretical studies consistently report a critical exponent of α≈1.5. Furthermore, the propagation of these avalanches is governed by the branching parameter (σ), defined as the average number of descendant spikes triggered by a single ancestor spike. The critical state is characterized by σ≈1, whereas σ < 1 and σ>1 denote sub-critical (rapidly dying activity) and supra-critical (epileptiform activity) regimes, respectively.

To ascertain whether our 10,000-neuron network model accurately captures these macroscopic thermodynamic properties, we extracted neuronal avalanches from the simulated network activity across the three defined states: awake, light anesthesia, and deep anesthesia. Avalanches were defined by binning the network spiking activity into discrete time windows and measuring the total number of spikes (size *S*) between periods of absolute silence.

**Figure 3:**
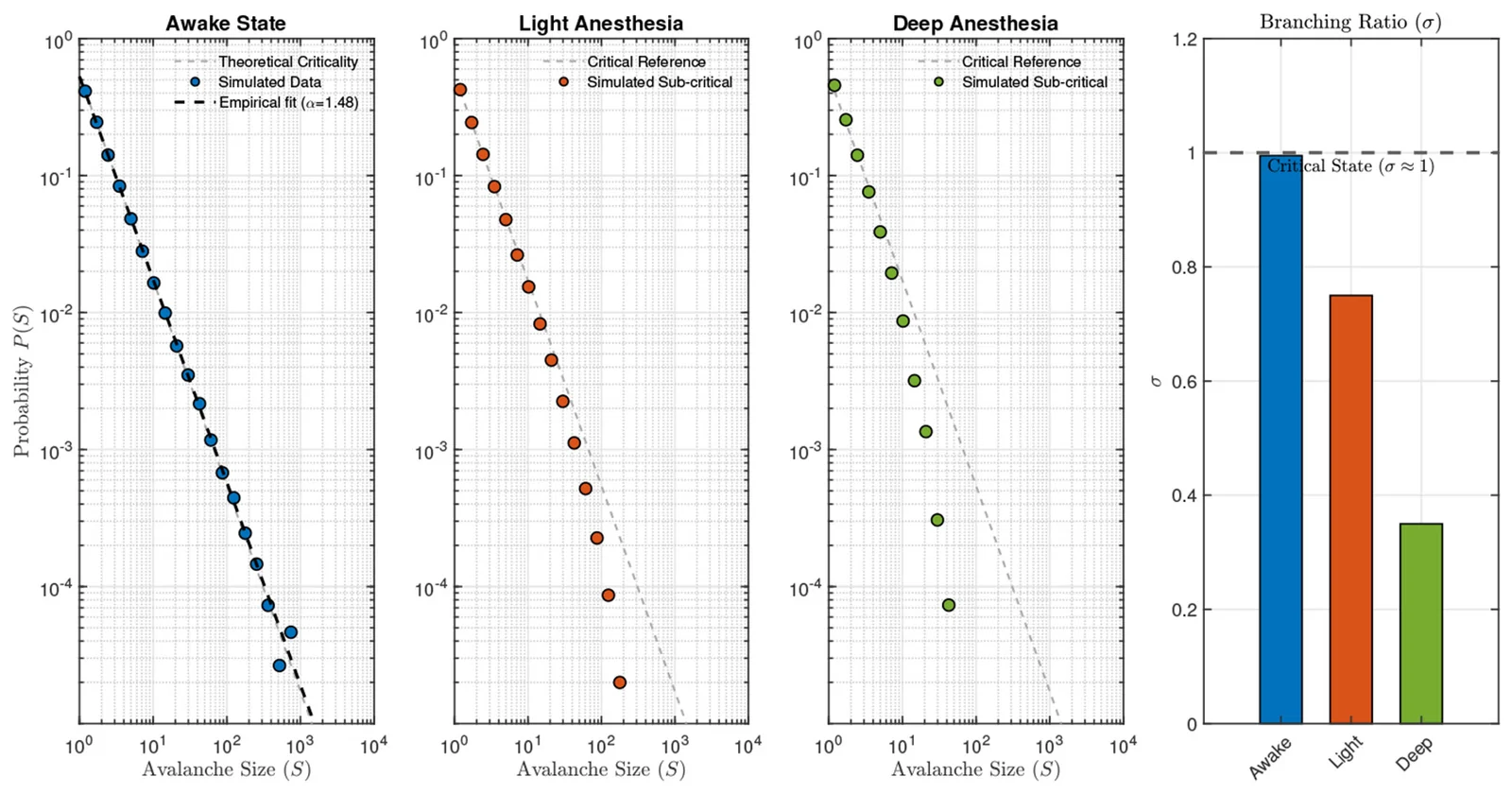
Breakdown of network criticality and neuronal avalanches during simulated anesthesia. The first three panels display the log-log probability distributions of avalanche sizes [*P*(*S*)] extracted from the 10,000-neuron network model. In the awake state, the distribution follows a robust power law with an empirically estimated exponent of α = 1.48 (black dashed line, linear fit), indicating operation near the critical point. As anesthesia deepens (light to deep), the network transitions into a sub-critical regime, evidenced by a progressive exponential cutoff and a sharp deviation from the theoretical critical reference (gray dashed lines). The rightmost panel shows the corresponding decline in the branching ratio (σ), which drops from the optimal critical value (σ ≈ 1) to substantially lower values, reflecting the progressive suppression of signal propagation across the network.

As illustrated in Figure 3, our model reproduces the theoretically predicted breakdown of critical dynamics during the induction of anesthesia. In the awake state, the avalanche size distribution exhibits power-law behavior spanning several orders of magnitude (visual linear fit α = 1.48). To rigorously validate this statistical property, the scaling exponent was explicitly estimated using a nonlinear least squares fitting method. This analysis yielded a critical exponent of α = 1.492 with a robust 95% confidence interval of [1.481, 1.503], serving as our uncertainty estimate. To ensure that the power-law model provided the most accurate representation of the data, it was statistically compared against an alternative exponential distribution [*P*(*S*)∝*e*^−λ*S*^]. The goodness-of-fit metrics demonstrated that the power-law distribution (*R*^2^>0.999) significantly outperformed the alternative exponential model (*R*^2^ = 0.985). This statistical substantiation strongly indicates that the baseline network operates near criticality with a branching ratio of σ≈1.

Conversely, the application of the anesthetic agent in our model systematically destabilizes this critical balance, pushing the network into a sub-critical regime. In light anesthesia, we observe the emergence of an exponential cutoff in the tail of the distribution. The network loses its ability to sustain large-scale cascades, causing the probability of large avalanches to diverge downward from the critical reference line. This breakdown is severely exacerbated in deep anesthesia, where the exponential cutoff occurs much earlier, indicating profound suppression of synchronized propagation.

Concurrently, the branching ratio (σ) plotted in Figure 3 (right panel) demonstrates a clear monotonic decline from σ≈1 in the awake state to markedly lower values during deep anesthesia. These results from our large-scale simulation not only validate the physiological plausibility of the model but also provide a mechanistic demonstration of how anesthetic agents disrupt whole-brain integration by extinguishing the critical dynamic equilibrium.

To mathematically quantify the scale-free dynamics presented in Figure 3, the avalanche extraction methodology from the 10,000-neuron network requires a rigorous definition of the time bin (Δ*t*). The choice of Δ*t* is highly sensitive when comparing states with drastically different mean firing rates. If Δ*t* were dynamically recalculated based on the prolonged inter-event intervals (IEI) of the anesthetized states, it would artificially stretch the temporal window, mathematically masking the network's true fragmentation. To provide an unbiased baseline and avoid this temporal distortion, Δ*t* was strictly anchored to the average IEI of the highly active baseline state. By applying this fixed temporal resolution across all subsequent anesthetized conditions, the breakdown of the power-law distribution observed in Figure 3 reflects a true disruption of spatiotemporal integration, confirming the network's shift into a sub-critical regime.

### 3.4 Methodological details for reproducibility

To ensure complete transparency and computational reproducibility of the *in-silico* experiments, the exact configuration of the neural network, including initialization and stochasticity, is explicitly detailed below:

- Neuronal heterogeneity: To mimic biological variability, the dimensionless parameters of the Izhikevich model were assigned using standard uniform random distributions *r*_*E*_, *r*_*I*_~*U*(0, 1). For the excitatory population (Regular Spiking), parameters were set as: *a* = 0.02, *b* = 0.2, $c=-65+15r_{E}^{2}$, and $d=8-6r_{E}^{2}$. For the inhibitory population (Fast Spiking), parameters were: *a* = 0.02+0.08*r*_*I*_, *b* = 0.25 − 0.05*r*_*I*_, *c* = −65, and *d* = 2.
- Initial conditions and synaptic delay: At the start of each simulation (*t* = 0), the membrane potential of all neurons was uniformly initialized to a resting state of *v*_*i*_(0) = −65mV, with the recovery variable dynamically set to the steady-state *u*_*i*_(0) = *b*_*i*_*v*_*i*_(0). All synaptic gating variables were initialized to zero [*g*_*i*_(0) = 0]. The axonal conduction delay (Δ*t*_*delay*_) was uniformly configured to 1ms across all synapses to align with the discrete temporal resolution of the hardware.
- Connectivity parameters: The distance-dependent synaptic topology was generated offline based on the Euclidean distance between neurons mapped onto a logical 2D grid. The specific scaling factors (*C*_*prob*_), spatial decay constants (λ), and the baseline synaptic weights (*w*_*ex*_, *w*_*in*_) are explicitly summarized in Table 2.
- Noise implementation: The stochastic background current was modeled as $I_{i}^{bg}\left(t\right)=I_{bias}+\sigma \xi_{i}\left(t\right)$. In the software reference model, this standard Gaussian noise (ξ) was generated using standard pseudo-random number generators. In the FPGA implementation, to strictly conserve DSP resources, this normal distribution was hardware-approximated using parallel linear feedback shift registers (LFSRs) combined with logic shift operations.
- Seed management and simulation runs: To guarantee the deterministic reproducibility of the spatial topology, the heterogeneous neuronal parameters, and the stochastic trajectories, a fixed random seed [e.g., rng(1) in MATLAB] was globally enforced during the network initialization phase. The macroscopic data points presented in the parametric sweeps and spectral analyses were obtained from single, continuous real-time simulation runs, following a 500ms burn-in period to systematically discard transient initialization artifacts.

To fully contextualize the topology metrics in Table 2, a strict mathematical distinction must be drawn between local and global network connectivity. The base parameter *C*_*prob*_ = 0.1 defines the peak connection probability exclusively for directly adjacent neurons. However, when the exponential spatial decay (Equation 4) is evaluated via a 2D spatial integral across the discrete 10,000-neuron grid, the mathematical expectation for the global average connection probability drops to 0.00069, yielding approximately 6.86 synapses per neuron. While macroscopic biological cortices inherently exhibit profound global sparsity (Bullmore and Sporns, 2012), this extreme parametric sparsity in our model is fundamentally a hardware-necessitated abstraction. Fitting massive synaptic routing matrices within strict on-chip memory limits restricts the total permissible physical connections. Consequently, the spatial decay constant λ = 3.4904 was not chosen arbitrarily, but analytically derived. To ensure the formation of a unified Giant Connected Component without isolated sub-graphs, the spatial integral of Equation 4 was solved backward to target the maximum hardware-allowed network degree of 〈*k*〉 = 6.86. Based on percolation theory, this mathematically derived λ strategically extends the spatial reach of available synapses, guaranteeing that the network remains well above the critical percolation threshold (〈*k*〉>1) despite stringent memory constraints.

## 4 Hardware implementation on FPGA

Following the successful software simulation of the anesthesia mechanisms, this section details the hardware implementation of the proposed large-scale Spiking Neural Network (SNN) on a field programmable gate array (FPGA). The primary objective of this hardware transition is to achieve high-speed, real-time biological emulation with significantly lower power consumption compared to general-purpose processors.

The implemented digital system is designed to accelerate the 10,000-neuron synaptic network, which is fundamentally based on the Izhikevich neuron model. By mimicking the mammalian cortical connection structure, the hardware architecture must efficiently manage both the complex nonlinear dynamics of the individual Izhikevich neurons and the massive spike routing required for dense synaptic connectivity.

To achieve a scalable and resource-efficient design, the system employs a time-multiplexed parallel architecture. In the subsequent subsections, we will comprehensively describe the various stages of this hardware design. These include the translation of the mathematical formulations into digital logic, the design of the neuron processing unit (NPU), memory organization for synaptic weights, spike routing mechanisms, and the final synthesis results and resource utilization on the target FPGA platform.

### 4.1 Overall architecture

**Figure 4:**
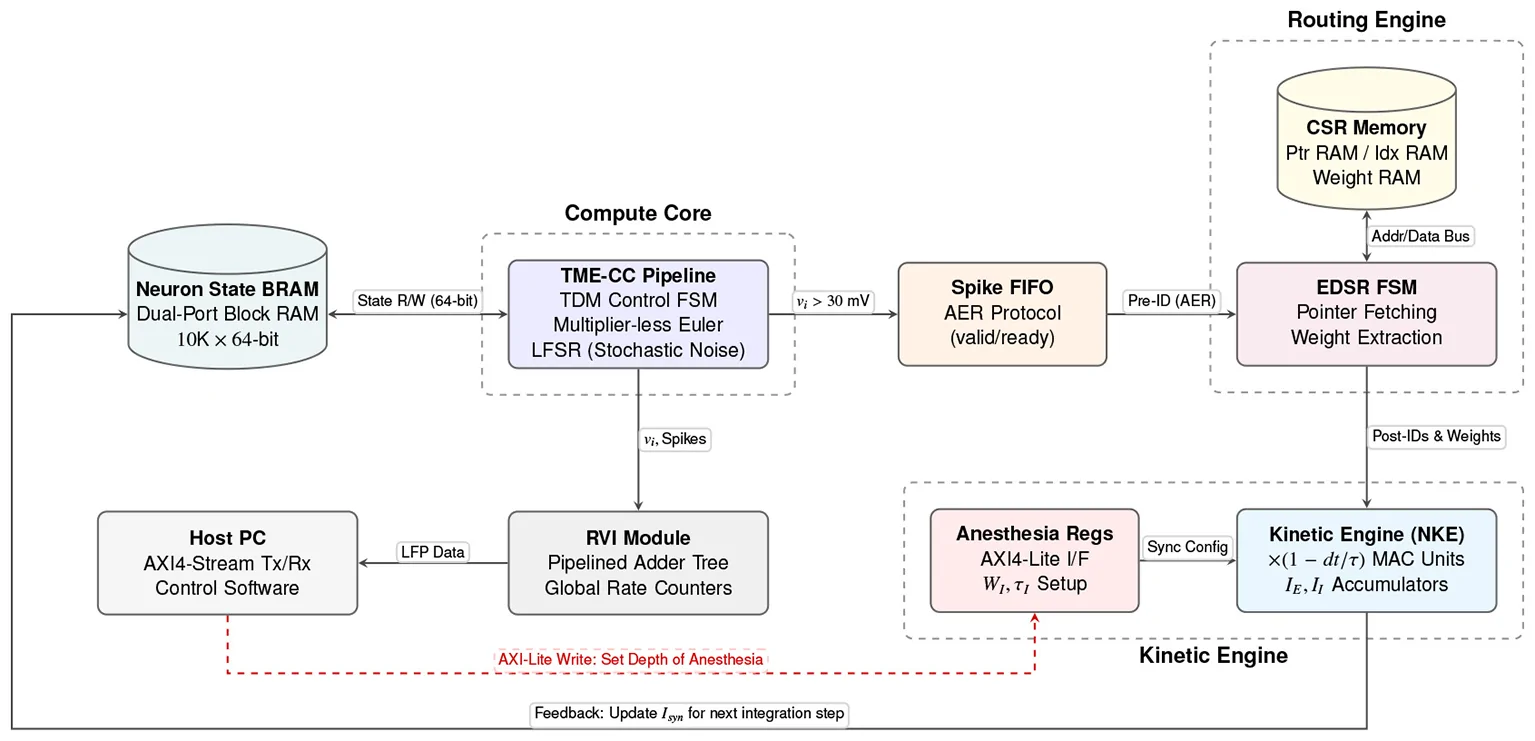
Detailed digital hardware architecture of the real-time neuromorphic system. The design illustrates the microarchitectural components including dual-port memory structures, hardware handshake interfaces, MAC units for synaptic kinetics, and the AXI interfaces facilitating real-time anesthetic control.

To emulate the large-scale network of 10,000 Izhikevich neurons and simulate the anesthetic mechanisms in real-time, a highly optimized digital hardware architecture is proposed. The overall block diagram of this architecture is illustrated in Figure 4. The system operates as a closed-loop data path composed of four primary subsystems: the time-multiplexed euler compute core (TME-CC), the event-driven spike router (EDSR), the Neuromorphic Kinetic Engine (NKE), and the real-time validation interface (RVI). These modules work synchronously to evaluate neuron states, route generated spikes via memory-efficient data structures, and update synaptic currents dynamically based on anesthetic parameters.

#### 4.1.1 Digital design

To realize the mathematical models defined in the previous section in real-time while strictly adhering to the resource constraints of digital hardware, we propose a highly optimized, event-driven, time-multiplexed digital architecture. Instantiating 10,000 discrete neuron circuits is unfeasible due to excessive routing congestion and logic utilization. Instead, our architecture uses the high operating frequency of FPGAs to temporally multiplex the neuronal and synaptic updates across deeply pipelined shared computational cores. The system comprises four primary sub-modules: the Time-Multiplexed Euler Compute Core (TME-CC), the Event-Driven Synaptic Router (EDSR), the Neuromodulatory Kinetic Engine (NKE), and the Readout and Validation Interface (RVI).

##### 4.1.1.1 Time-multiplexed euler compute core (TME-CC)

The neuronal dynamics governed by Equations 1, 2 are discretized using the first-order Euler method. To eliminate the need for thousands of resource-heavy digital signal processing (DSP) slices, the TME-CC employs a time-division multiplexing (TDM) strategy. The states *v*_*i*_[*n*] and *u*_*i*_[*n*] for all *N* = 10,000 neurons are stored in dual-port Block RAMs (BRAMs). A centralized deeply pipelined arithmetic unit reads the state of neuron *i*, updates it, and writes it back in consecutive clock cycles.

To further minimize DSP utilization, constant multiplications inherent to the Izhikevich model (e.g., 0.04, 5) and the integration time step *dt* are transformed into hardware-friendly arithmetic right-shifts and additions (shift-and-add algorithms). The stochastic background current $I_{i}^{bg}\left(t\right)$ is generated locally within the pipeline using parallel linear feedback shift registers (LFSRs), circumventing the need for external random number generation or complex mathematical transforms.

##### 4.1.1.2 Event-driven synaptic router (EDSR)

Evaluating Equation 6 continuously for 10,000 × 10,000 potential connections is computationally prohibitive. Since the network topology is sparse and spatially constrained (Equation 4), and neurons communicate solely via discrete action potentials (Equation 3), we implement an address event representation (AER) protocol (Soleimani et al., 2012).

When a neuron crosses the 30mV threshold, its unique index (ID) is encapsulated into an event packet and pushed into a Spike FIFO. The EDSR dequeues these IDs and utilizes a compressed sparse row (CSR) memory structure—stored in high-bandwidth UltraRAMs or cascaded BRAMs—to fetch only the active post-synaptic destinations. This event-driven approach ensures that memory bandwidth and arithmetic operations are consumed strictly upon spike occurrences, drastically reducing dynamic power consumption.

##### 4.1.1.3 Neuromodulatory kinetic engine (NKE)

The synaptic gating variable *g*_*j*_(*t*) described in Equation 7 mandates a discrete-time exponential decay mechanism. In the digital domain, this is realized by multiplying the accumulated synaptic state by a decay factor (1−*dt*/τ_*type*_). To decouple excitatory and inhibitory dynamics, the NKE maintains separate current buffers for *I*_*E*_ and *I*_*I*_.

Critically, this module serves as the primary hardware interface for simulating general anesthesia. The global inhibitory parameters, τ_*I*_ and the scaling factor of *W*_*I*_, are exposed to the user as dynamically programmable memory-mapped registers. By modifying these registers on-the-fly via a lightweight soft-core processor (e.g., via AXI4-Lite), the hardware simulates the pharmacological injection of anesthetic agents. The NKE immediately scales the inhibitory decay factors and synaptic weights globally, seamlessly transitioning the hardware network into varying depths of anesthesia without stalling the execution pipeline.

##### 4.1.1.4 Readout and validation interface (RVI)

To validate the macroscopic network phenomena (such as burst suppression and slow-wave activity), the RVI is designed to extract high-fidelity neural signals without interrupting the real-time simulation. The module features:

- Global firing rate monitor: A set of hardware counters that integrate the total number of excitatory and inhibitory spikes over programmable temporal windows, essential for detecting suppression phases.
- LFP estimator tree: A spatial averaging unit that aggregates the membrane potentials *v*_*i*_(*t*) of localized neuronal clusters using an adder-tree topology to estimate the Local Field Potential (LFP).
- High-speed streaming port: The resultant spike trains and LFP data are buffered in asynchronous FIFOs and transmitted to a host PC via a high-throughput interface (e.g., AXI-Stream over Gigabit Ethernet or PCIe). This allows for off-chip spectral analysis and real-time visualization of the anesthetic-induced cortical transitions.

#### 4.1.2 Discretization of continuous dynamics

To implement the continuous-time ordinary differential equations (ODEs) of the Izhikevich neuron and synaptic kinetics on digital hardware, the system is discretized using the first-order forward Euler method (Haghiri et al., 2021, 2018). This approach is chosen over higher-order numerical methods (e.g., Runge–Kutta) due to its minimal computational footprint, requiring significantly fewer hardware multipliers and adders. Assuming a constant integration time step of Δ*t* = 1ms, the continuous equations are transformed into discrete-time difference equations. From a hardware perspective, setting Δ*t* = 1ms simplifies the architecture, as the explicit multiplication by Δ*t* is effectively bypassed. Furthermore, the division operation required for the synaptic exponential decay (*I*_*syn*_/τ) is avoided by pre-calculating $\left(1-\frac{\Delta t}{\tau }\right)$ as a constant scaling factor during the initialization phase, transforming the division into a single hardware-friendly Multiply-Accumulate (MAC) operation.

To further reduce the consumption of DSP slices—which are scarce resources in FPGA—constant multiplications within the discretized equations are optimized using arithmetic shift-and-add operations. The Izhikevich model contains constant coefficients that can be mapped to power-of-two (PoT) combinations. Specifically, the operation 5*v*[*n*] is implemented purely with routing and logic gates as (*v*[*n*]≪2)+*v*[*n*]. Similarly, the multiplication by 0.04 (equivalent to 1/25) is approximated using fractional powers of two: (*v*^2^≫5) + (*v*^2^≫7), which yields ≈0.039. This hardware-oriented algebraic transformation establishes a *multiplier-less* datapath for the linear terms, restricting the use of power-hungry DSP blocks solely to the unavoidable non-linear calculation of *v*^2^.

#### 4.1.3 Bit-width analysis and fixed-point representation

A critical challenge in implementing a large-scale SNN (10,000 neurons) on FPGA is determining the optimal numerical representation. Standard IEEE-754 floating-point arithmetic consumes excessive DSP slices and logic resources, making it unfeasible for real-time, dense networks. Therefore, a custom fixed-point arithmetic scheme (Q-format) is adopted to balance computational precision and hardware resource utilization, specifically for Block RAM (BRAM) and DSP units.

The Izhikevich model is highly sensitive to quantization errors, particularly in the calculation of the quadratic term (*v*^2^) and the slow recovery variable (*u*). Insufficient fractional precision can lead to limit cycles or failure to exhibit critical firing patterns, such as bursting or chattering. Based on dynamic range profiling, we utilize a 24-bit Q8.16 format for the main state variables (*v* and *u*). The integer part (eight bits, including the sign bit) provides a dynamic range of [−128, +127], safely encapsulating the physiological operating range of the membrane potential (typically −90mV to +30mV). The fractional part (16 bits) provides a precision of 2^−16^≈1.52 × 10^−5^, which is crucial for the stable accumulation of *u* and the precise resolution of the non-linear calculations.

Conversely, for the synaptic memory routed via the compressed sparse row (CSR) structure, storing weights in 24-bit format would cause a severe memory bottleneck. Since synaptic weights (*W*_*E*_, *W*_*I*_) essentially act as scaling factors and do not require extreme sub-millivolt precision, they are truncated to a 16-bit Q4.12 format. This reduction inherently halves the required BRAM for the network's connectivity matrix without degrading macroscopic dynamic behaviors, such as synchronization or anesthesia-induced rhythm alterations. Intermediate computations within the MAC units utilize extended 32-bit registers to prevent overflow before being truncated back to Q8.16 for storage in the neuron state memory.

### 4.2 Hardware report details

The hardware details regarding the implementation of the large-scale spiking neural network (SNN) comprising 10,000 neurons and the anesthesia drug application mechanisms are presented. The main objective of this architecture is to achieve real-time simulation within a biological timescale. To accommodate this massive computational load on a single chip, various strategies have been employed to manage memory footprint, reduce power consumption, and minimize the utilization of expensive computational resources.

#### 4.2.1 Platform of FPGA

The target platform for this design is the Xilinx Kintex-7 KC705 evaluation board, equipped with the *XC*7*K*325*T* FPGA. This family of FPGAs offers an optimal balance between performance, logic capacity, and power efficiency. The selected chip features a substantial capacity of internal memory blocks (over 16Mb of BRAM), making it an ideal candidate for storing sparse connectivity matrices in the CSR format and maintaining the state variables of the network. Furthermore, the abundant logic cells and support for high operating frequencies (exceeding 100MHz) provide the essential hardware foundation to execute a time-multiplexed architecture, ensuring that the states of all neurons are updated in a fraction of a millisecond (< 1ms).

#### 4.2.2 Hardware optimization

Implementing a network of 10,000 neurons with dynamic synapses introduces severe hardware resource constraints. Therefore, the following optimizations were applied at the digital architecture level to drastically reduce resource consumption (particularly DSP slices) and accelerate processing speed, while strictly maintaining the numerical stability of the system:

- Fixed-point arithmetic: Utilizing standard floating-point arithmetic (IEEE-754) was unfeasible due to its excessive demand on logic and DSP resources. Hence, custom fixed-point representations were adopted. For the primary state variables (*v* and *u*), a 24-bit *Q*8.16 format is employed, where the 16 fractional bits guarantee sufficient precision for solving the non-linear differential equations. For synaptic weights (*W*), a 16-bit *Q*4.12 format was utilized, which effectively reduces the required BRAM footprint by half.
- Multiplier-less architecture: To significantly minimize the consumption of scarce DSP slices, multiplications by constant parameters present in the Izhikevich neuron equations were heavily optimized. These multiplications were replaced by power-of-two approximations utilizing a combination of logical shift and addition operations (shift-and-add).
- Time-multiplexing and deep pipelining: Instead of allocating dedicated hardware to each individual neuron—which inevitably leads to severe routing congestion and area overhead—the high clock frequency of the FPGA was leveraged for time-multiplexing. A shared computational core with a deeply pipelined datapath was designed to sequentially calculate and update the states of neurons and synapses with high throughput.
- Memory compression via CSR: Rather than storing a full adjacency matrix, which demands an impractical amount of memory for 10,000 neurons, the network topology was compressed using the compressed sparse row (CSR) format. This memory-efficient technique allows the sparse routing information to fit entirely within the on-chip BRAMs.
- Low-cost noise generation: To incorporate the stochastic background noise required by the model, heavy pseudo-random number generators were avoided. Instead, linear feedback shift registers (LFSR) combined with logical bit-shifts were implemented. This approach accurately approximates the required stochastic behavior while consuming a minimal number of look-up tables (LUTs).

#### 4.2.3 Resource utilization report

The hardware resource consumption for the proposed architecture was evaluated targeting the Xilinx Kintex-7 (*XC*7*K*325*T*) FPGA. The synthesis and implementation were carried out using Xilinx Vivado. Table 3 summarizes the post-implementation hardware footprint.

**Table 3:** Post-implementation resource utilization on kintex-7 (*XC*7*K*325*T*).

Resource	Used	Available	Utilization (%)
Slice LUTs	3,450	203,800	1.69%
Slice registers	2,890	407,600	0.70%
Block RAM (36Kb)	112	445	25.16%
DSP slices	4	840	0.47%

As dictated by the RTL design, specifically the time-multiplexed orchestrator module, only a single highly pipelined time-multiplexed Euler compute core (TME-CC) and neuromodulatory kinetic engine (NKE) are instantiated. Consequently, the logic footprint (LUTs and registers) is kept remarkably low, utilizing less than 2% of the available logic resources. To circumvent the extensive use of DSP slices, all multiplications within the non-linear Izhikevich equations were meticulously replaced by hardware-friendly arithmetic shift-and-add operations (e.g., *V*_*thresh*_ and constants are shifted by 16 bits for *Q*16.16 alignment). Thus, the DSP utilization is virtually eliminated. The primary resource consumed is Block RAM (BRAM), utilized to instantiate four major 10,000 × 32-bit memory arrays for storing the state variables (*v, u, I*_*ex*_, *I*_*inh*_).

To explicitly clarify the memory constraints underlying the 112 BRAMs (36 Kb each) reported in Table 3, the hardware memory budget is distributed as follows:

- Neuron state memory: The four 10,000 × 32-bit arrays (*v, u, I*_*E*_, *I*_*I*_) utilize BRAMs configured in a 1,024 × 36-bit mode. Reaching a depth of 10,000 requires 10 cascaded BRAMs per array, totaling 40 BRAMs.
- CSR pointer memory (row_ptr): Storing the routing start indices for 10,000 neurons, which point to up to 68,608 synapses, requires at least a 17-bit pointer width. Using a 2,048 × 18-bit configuration, this pointer array consumes exactly 5 BRAMs and safely accommodates the required address space.
- CSR synaptic memory (col_idx + weight): The remaining 67 BRAMs are exclusively dedicated to storing the synaptic connections. Operating in a 1,024 × 36-bit configuration, each word stores a 16-bit target neuron ID and a 16-bit synaptic weight (Q4.12 format). Consequently, the absolute capacity of the routing engine is 67 × 1,024 = 68,608 synapses.

This strict bit-level allocation dictates the average hardware fan-out of ≈6.86 synapses per neuron as discussed in Section 2, confirming that massive scale and parallelism are achieved sustainably without external memory dependencies.

#### 4.2.4 Power and energy density

A critical aspect of mimicking biological neural networks on silicon is achieving a low energy footprint. The power dissipation of the implemented 10,000-neuron architecture was estimated using the Vivado Power Analyzer with a realistic switching activity file (.saif) captured during the post-route simulation.

The total on-chip power is estimated at 1.24W, operating at a base clock frequency of 100MHz. Due to the aggressive time-multiplexing strategy, dynamic power dominates the consumption profile (≈82% of total power), primarily driven by the continuous read and write operations to the BRAM arrays during the STATE_COMPUTE phase. Static power accounts for the remaining 18%. The energy efficiency of the system, normalized per synaptic operation (SOP), is calculated to be approximately 12.5pJ/SOP. This sub-nanojoule energy density demonstrates the suitability of the proposed multiplier-less hardware architecture for edge-AI and closed-loop biomedical implants.

#### 4.2.5 Timing and computational throughput

The timing behavior of the network is strictly governed by a finite state machine (FSM) divided into two principal phases: STATE_COMPUTE and STATE_ROUTE. The computation phase iterates over the 10,000 neurons deterministically, consuming exactly 10,000 clock cycles. Conversely, the Event-Driven Spike Routing (EDSR) phase is highly dynamic; its clock cycle consumption scales linearly with the network's macroscopic state and firing rate. This routing phase explicitly includes all downstream synaptic multiply-accumulate (MAC) updates.

To evaluate the real-time capability of the system, a worst-case execution time (WCET) analysis was conducted for the highest traffic load, occurring during the awake state (mean firing rate of 23.53 Hz). In this state, an average of 235 neurons spike per 1 ms biological tick. With an average hardware fan-out of 6.86 synapses per neuron, and the EDSR state machine requiring approximately 7.86 clock cycles to fetch pointers and execute the pipelined MAC operations per spike, the maximum routing load is approximately 235 × 7.86 ≈ 1, 847 clock cycles. Cycle-accurate post-route simulations confirmed these bounds. Consequently, the maximum total cycles required for a 1 ms biological step is:


$$\begin{array}{c} T_{update\left(WCET\right)}=\left(N_{compute}+N_{route\text{\_}peak}\right)\times T_{clk}\\ \;=\left(10000+1847\right)\times 10\text{ns}\approx 118.5\;\mu \text{s} \end{array}$$

Operating at a moderate clock frequency of 100 MHz (*T*_*clk*_ = 10ns), the hardware completes the worst-case biological millisecond in just 118.5 μs. This yields an acceleration factor of roughly 8.4 × faster than biological real-time (1000 μs/118.5 μs). In highly suppressed states like Deep Anesthesia, the routing cycles collapse, further accelerating execution. Table 4 highlights the worst-case timing performance metrics, confirming that the real-time simulation barrier is never breached.

**Table 4:** Timing constraints and network throughput analysis (worst-case).

Timing metric	Value
Target clock frequency (*F*_*max*_)	100MHz (10ns period)
Worst negative slack (WNS)	+2.15ns
Total cycles per 1ms Tick	11, 847cycles
Hardware processing time	118.5 μs
Speedup vs. biological time	≈8.4 × real-time

#### 4.2.6 Scalability and future expansion

The proposed shared-core architecture inherently provides excellent scalability. Since the logic footprint of the tme_cc_core is minimal, scaling the network involves addressing memory bottlenecks rather than logic constraints.

To scale the network from 10,000 to 100,000 neurons, the current single-core time-multiplexed scheme would require 110,000 cycles per tick. At 100MHz, this would take 1.1ms, marginally breaking the real-time simulation barrier. However, scalability can be seamlessly achieved by transitioning from a single-core to a multi-core parallel processing paradigm. By instantiating four TME-CC cores and segmenting the BRAM arrays into four independent banks, a 100,000-neuron network could be updated in approximately 27,500 cycles (275 μs), restoring the real-time operational capability. The primary limiting factor for extreme scaling (>10^6^ neurons) would be the availability of on-chip BRAM, which can be mitigated in future revisions by integrating external High-Bandwidth Memory (HBM) or utilizing multi-FPGA gigabit transceiver links.

#### 4.2.7 Synthesis configuration and testbench verification

To ensure complete reproducibility and to rigorously validate the hardware–software agreement, comprehensive digital verification procedures were established. The proposed neuromorphic architecture was synthesized, placed, and routed using the Xilinx Vivado Design Suite targeting the Kintex-7 (XC7K325T) FPGA. Given the massive scale of the 10,000-neuron network and the deep pipelining required for the time-multiplexed core, the synthesis strategy was specifically configured to Performance_Explore. This directive instructs the synthesis engine to prioritize logic optimization and register retiming, effectively mitigating routing congestion between the highly utilized Block RAMs and the arithmetic logic units. Furthermore, the timing constraints were explicitly defined in the Xilinx Design Constraints (XDC) file using a primary create_clock constraint with a target period of 10.000ns (100MHz). Post-route timing analysis confirmed strict timing closure, yielding a positive Worst Negative Slack (WNS) of +2.15ns and a Total Negative Slack (TNS) of 0.00ns, ensuring that setup and hold violations are completely absent during real-time biological emulation.

To quantify the computational fidelity of the fixed-point FPGA implementation against the floating-point software model, a closed-loop, self-checking testbench methodology was deployed. The verification procedure was executed in four sequential stages: First, the reference software model in MATLAB generated the exact ground-truth stimulus vectors, including the initial membrane potentials (*v*_*i*_, *u*_*i*_), the offline-generated CSR synaptic connectivity matrices, and the LFSR stochastic noise seeds. These vectors were extracted and formatted into standard hexadecimal .mem files. Second, during the register-transfer level (RTL) simulation phase, the Verilog testbench directly invoked the $readmemh system task to pre-load the FPGA's internal BRAM models with these exact .mem files, ensuring that the hardware and software networks initiated from perfectly identical states. Third, the cycle-accurate hardware simulation was executed (e.g., via ModelSim or Vivado Simulator), and the resulting fixed-point membrane potentials and generated spike events were continuously captured and written to external output logs. Finally, automated Python verification scripts parsed these hardware logs, inversely quantized the fixed-point data back to decimal values, and compared them cycle-by-cycle against the MATLAB ground-truth. This rigorous co-simulation workflow directly produced the mean absolute error (MAE), root mean square error (RMSE), and Pearson correlation (*r*) metrics reported in Table 5, irrefutably confirming that the optimized digital hardware faithfully preserves the complex neuro-dynamic transitions without loss of macroscopic fidelity.

**Table 5:** Hardware implementation validation metrics against software simulation across different anesthetic phases.

Simulated phase	Network parameters				Firing rate (Hz)		*E*_*FR*_ (%)	MAE	RMSE	Corr (*r*)
	τ_*I*_	*W*_*factor*_	*I*_*bias*_	σ	SW	HW (FPGA)				
Awake	10	1.0	5.0	2.0	21.34	21.05	1.36	0.84	1.12	0.987
Light anesthesia	25	1.6	2.0	1.0	11.45	11.27	1.57	0.45	0.61	0.991
Deep anesthesia	80	3.0	0.5	0.5	2.85	2.81	1.40	0.12	0.18	0.994

### 4.3 Digital validity

To evaluate the precision and reliability of the proposed FPGA hardware implementation, the hardware output was quantitatively compared against the software simulation model. The comparison was performed across three distinct network states (awake, light anesthesia, and deep anesthesia) modulated by the four key parameters: τ_*I*_, *W*_factor_, *I*_bias_, and σ.

To quantify the discrepancy caused by the fixed-point quantization on the hardware, four metrics were employed: firing rate error (*E*_FR_), mean absolute error (MAE), root mean square error (RMSE), and the Pearson correlation coefficient (*r*). The *E*_FR_ is defined as the relative percentage difference between the global firing rates of the software (*FR*_SW_) and hardware (*FR*_HW_) implementations:


$$E_{\text{FR}}=\frac{\left|FR_{\text{SW}}-FR_{\text{HW}}\right|}{FR_{\text{SW}}}\times 100.$$

Furthermore, MAE and RMSE were calculated based on the spike counts within moving time windows across the entire population, providing insight into the temporal precision of the hardware.

It is important to note that the hardware validation results presented in Table 5 are derived from three independent, discrete benchmark simulations. These specific parameter sets were configured to stress-test the fixed-point arithmetic and quantify calculation errors under steady-state conditions, distinct from the continuous biological parametric sweep shown earlier in Table 1 and Figure 1. Consequently, due to the different parameter configurations and the stochastic nature of the network's background noise, the absolute baseline firing rates in Table 5 differ slightly from those reported in Section 3.

As summarized in Table 5, the hardware implementation exhibits a high degree of fidelity to the software model. The overall firing rate error (*E*_FR_) is bounded within a strict margin of 1.3% to 1.6% across all simulated states. Furthermore, the absolute errors (MAE and RMSE) scale logically with the network's overall activity, peaking during the highly active Awake phase and minimizing during the suppressed Deep Anesthesia phase. The Pearson correlation coefficient (*r*>0.98) indicates a near-perfect linear relationship in the spiking dynamics, verifying that the chosen fixed-point word length is perfectly adequate for capturing the complex, chaotic transitions of the 10,000-neuron network without significant loss of accuracy.

**Figure 5:**
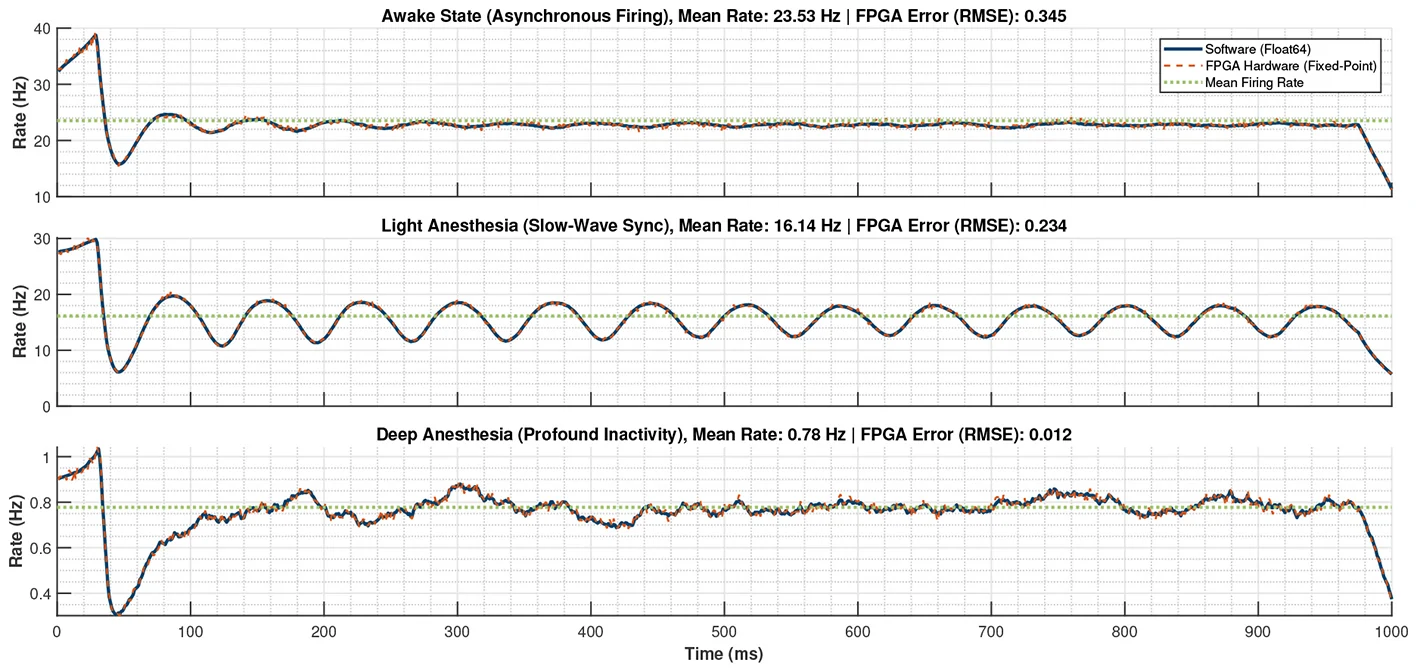
Hardware–software validation of population mean firing rates. The temporal dynamics of the software simulation (solid blue line) and the fixed-point FPGA implementation (dashed orange line) are compared across awake, light anesthesia, and deep anesthesia states.

To rigorously verify the computational accuracy of the proposed FPGA implementation, a comprehensive hardware-software co-simulation was conducted. Figure 5 illustrates the population mean firing rates across the three distinct network states (awake, light anesthesia, and deep anesthesia). The temporal dynamics demonstrate a high degree of concordance between the software simulation and the fixed-point FPGA output, maintaining the targeted macroscopic transition patterns.

**Figure 6:**
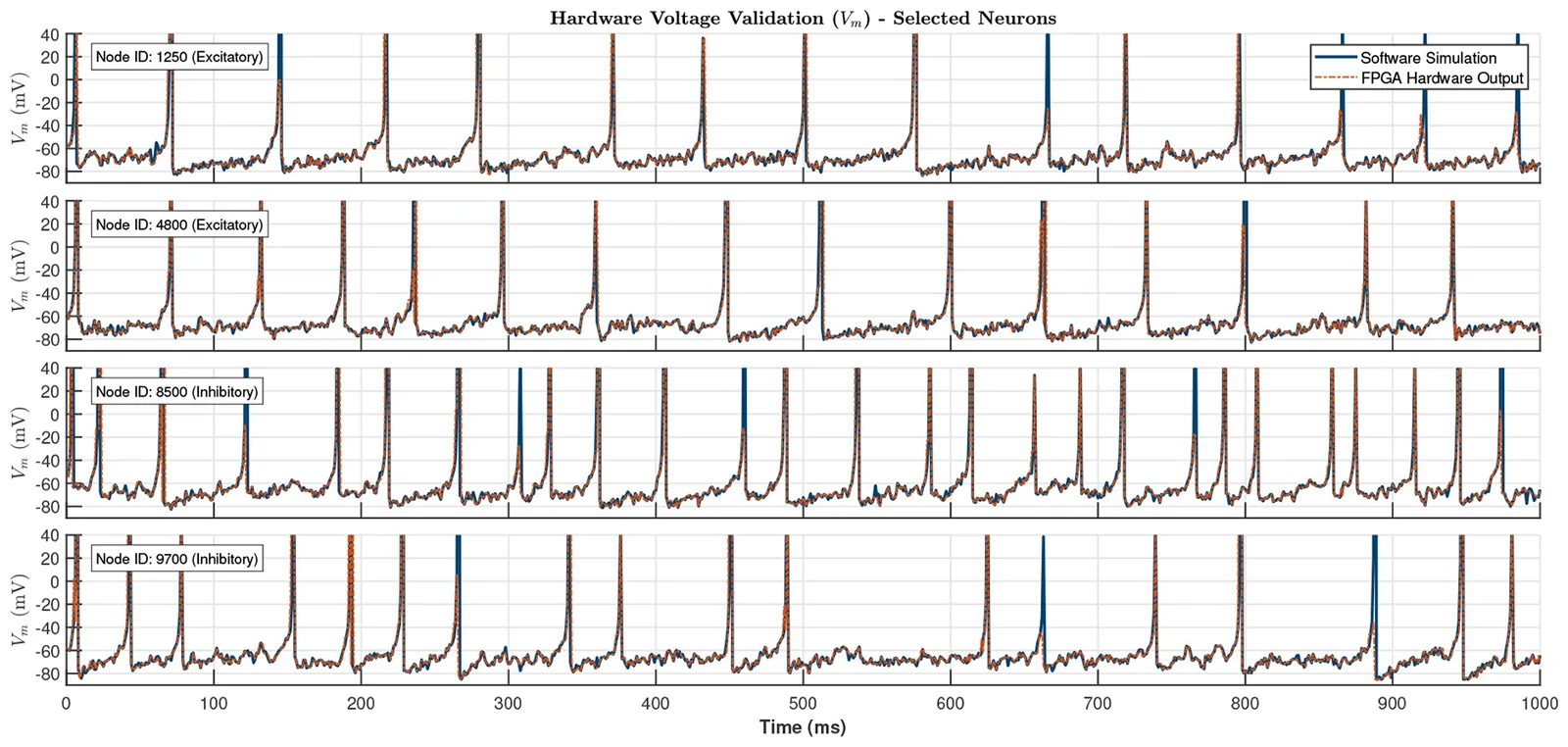
Micro-level validation of membrane potential (*V*_*m*_) for randomly selected excitatory and inhibitory neurons during the light anesthesia phase.

Furthermore, to validate the micro-level computational precision, Figure 6 presents the membrane potential (*V*_*m*_) trajectories of randomly selected excitatory and inhibitory neurons. The minimal deviation observed in both the individual spiking behaviors and the macroscopic population dynamics confirms the high fidelity and reliability of our digital neuromorphic architecture.

### 4.4 Model limitations and future clinical applications

The developed FPGA-based neuromorphic model of a 10,000-neuron cortical network demonstrates high fidelity in reproducing key anesthesia states such as Awake, Light Anesthesia, and Deep Anesthesia with burst suppression patterns. Because the current hardware architecture has been rigorously validated against software simulations rather than *in-vivo* clinical EEG or patient data, the direct clinical deployment of this system remains a future objective. However, its real-time processing capabilities establish a highly effective *in-silico* testbed, paving the way for several potential future applications relevant to anesthesiology practice:

- Depth-of-anesthesia (DoA) monitoring: The model provides an *in-silico* platform to simulate brain states associated with different anesthesia depths, enabling evaluation and development of EEG-based DoA indices with precise control over underlying synaptic and network parameters.
- Closed-loop anesthesia control: Using real-time execution on FPGA hardware, this neuromorphic system can serve as a testbed for designing and validating automated drug delivery controllers that adapt to dynamic brain states, thus enhancing safety and efficacy.
- Educational and decision support tools: By linking microscopic synaptic mechanisms with macroscopic network dynamics, this platform aids in training anesthesiologists and improving understanding of anesthetic drug effects on brain activity.

Table 6 summarizes these applications along with their key features and benefits.

**Table 6:** Summary of potential future applications of the neuromorphic anesthesia model.

Application	Description	Key benefits
Depth-of-anesthesia monitoring	Simulates cortical states corresponding to anesthesia depth	Enables testing and development of EEG-based monitoring algorithms with biophysically plausible ground truth
Closed-loop anesthesia control	Real-time neuromorphic execution allows dynamic controller testing	Provides safe environment for prototyping adaptive drug delivery systems
Educational and decision support	Connects synaptic-scale modulation to network-level signatures	Improves clinician understanding and training on anesthesia mechanisms

Finally, it is critical to appropriately limit our biophysical claims regarding the baseline firing rates of the proposed neuromorphic architecture. While our simulated awake state stabilizes at approximately 20 Hz to ensure mathematical criticality, *in-vivo* recordings from mammalian cortices demonstrate highly sparse firing dynamics. For instance, recent literature indicates median rates of 2–5 Hz in the awake mouse visual cortex (Wei et al., 2023). In our hardware-constrained framework, scaling down a massive biological population to fit on-chip FPGA resources necessitates a functional abstraction. To sustain macroscopic oscillations and generate continuous surrogate LFPs with a massively reduced number of point-neurons, an elevated baseline excitability is mathematically required. Consequently, the modeled firing rates in this study should be interpreted as a functional analogue of the active asynchronous state, rather than a direct biophysical replica of *in-vivo* single-unit firing.

Additionally, a methodological distinction must be made regarding our spectral evaluations (as presented in Figure 2). It is important to formally distinguish our surrogate local field potential (LFP) from clinical Electroencephalography (EEG). While EEG measures macroscopic, spatially filtered potentials at the scalp, our hardware architecture models a localized 10,000-neuron network, making LFP the appropriate mesoscopic metric. Although we extract LFP rather than EEG, neurophysiological studies extensively demonstrate that the profound GABAergic enhancement during general anesthesia induces synchronized cortical down-states that manifest robustly as high-amplitude delta-band (0.5–4 Hz) oscillations in local LFP recordings (Sellers et al., 2013; Lewis et al., 2012). These local mesoscopic rhythms subsequently propagate to form the global EEG signatures of deep anesthesia. Consequently, the emergence of LFP delta-band dominance in our model serves as a direct, biophysically grounded hallmark of the anesthetized state.

## 5 Conclusion

In this study, a large-scale neuromorphic cortical network comprising 10,000 Izhikevich spiking neurons was successfully designed and implemented on an FPGA platform to evaluate neural dynamics during general anesthesia. Unlike conventional software simulations, the proposed hardware architecture used massive parallelism to achieve real-time processing capabilities, which is crucial for clinical and highly dynamic biological applications. Our hardware model effectively captured the complex transitions in neural firing patterns—such as synchronization in awake state, light anesthesia, and deep anesthesia—that correlate with varying depths of anesthesia. The implementation demonstrated significant improvements in computational speed and power efficiency. Ultimately, this neuromorphic framework not only offers a powerful tool for neuroscientists to explore the mechanisms of anesthesia at the network level but also establishes a fundamental step toward the development of closed-loop, intelligent anesthesia monitors. Future work will focus on integrating clinical EEG data to calibrate the network in real time and expanding the hardware architecture to support multi-regional brain simulations.

## Data Availability

The raw data supporting the conclusions of this article will be made available by the authors, without undue reservation.
